# Exploring the Impact of Sports Participation on Social Capital and Health-related Factors in Individuals with Spinal Cord Injury: A Cross-sectional Study

**DOI:** 10.1298/ptr.E10295

**Published:** 2024-10-02

**Authors:** Kazuki KANEDA, Noriaki MAEDA, Takumi NAGAO, Ayano ISHIDA, Tsubasa TASHIRO, Makoto KOMIYA, Yukio URABE

**Affiliations:** 1 Graduate School of Biomedical and Health Sciences, Hiroshima University, Japan

**Keywords:** Spinal cord injury, Sports participation, Social capital, Physical activity, Psychological well-being

## Abstract

Objective: This study examined the impact of sports participation on the health status of individuals with spinal cord injuries (SCI), with emphasis on the role of health-related social capital (HRSC). Methods: This study included 65 individuals with SCI (42 who participated in sports and 23 who did not). The following information was obtained from the participants through an online questionnaire: their basic information, information regarding activities of daily life independence, physical activity, mental health, lifestyle, insomnia, and social capital. We compared the outcomes between participants with and without sports participation and examined their correlations. Multiple regression analysis with forced entry was performed to determine the association between HRSC and health outcomes. Results: Physical activity, mental health, and HRSC were significantly higher in individuals with SCI who participated in sports (p <0.01 or p <0.05) than in individuals with SCI who did not participate in sports. The HRSC of individuals with SCI with sports participation showed a significant positive correlation with lifestyle and a significant negative correlation with insomnia score (p <0.05). Multiple regression analysis revealed that higher HRSC was associated with lifestyle in individuals with SCI who participated in sports (p <0.05) compared with individuals with SCI who did not participate in sports. Conclusion: The study findings underscore the potential benefits of sports participation in individuals with SCI, including increased physical activity and development of HRSC. However, it is essential to consider the implications of sports involvement on the psychological well-being of individuals with disabilities and provide appropriate support.

## Introduction

Spinal cord injuries (SCI) profoundly impact individuals, causing varying degrees of motor, sensory, and autonomic dysfunctions^1)^. Depending on the level and severity of injury, individuals may experience partial or complete paralysis, loss of sensation, impaired mobility, and a range of secondary health complications^2^^,^^3)^. These triggers have various physical, psychological, and lifestyle effects on individuals^4^^,^^5)^.

Engaging in sports activities has been recognized as a potential means of promoting overall improvements in physical activity, mental health, and lifestyle^6^^,^^7)^. This is expected to be true for people with disabilities, including those with SCI^8)^. In addition, the concept of health-related social capital (HRSC) has been recognized as an important determinant of health outcomes, which encompasses the resources, support, and networks available to individuals through their personal and social connections^9)^. Sports participation has been reported to be effective in building HRSC, and individuals who participate in sports activities become part of a community of teams, peers, coaches, and supporters, providing opportunities to develop social interactions and relationships^10)^. Previous studies examining the effects of participation in sports for individuals with SCI have reported positive effects on physical activity, mental health, and lifestyle^11^^–^^13)^. Scattered reports on interpersonal relationships also show that they enhance current connections with family and friends^13^^,^^14)^. Promoting community interaction through sports participation is expected to build social capital and positively impact factors related to physical and mental health and lifestyle factors. However, despite the growing interest in the potential benefits of sports participation and HRSC, research investigating the relationship between these factors in individuals with SCI remains scarce. Examining how social capital building and health outcomes are impacted through sports participation among individuals with SCI is a new perspective and could further the benefits of sports participation.

This study aimed to investigate the impact of sports participation on the health status of individuals with SCI by comparing the characteristics of individuals with SCI who did and did not participate in sports, and the results of social capital, physical activity level, psychological well-being, and other related indicators.

## Methods

### Study participants

This study included a total of 65 individuals with SCI (42 who participated in sports: 42.5 ± 10.2 years old, 40 males, 2 females; 23 who did not participate: 41.6 ± 8.5 years old, 21 males, 2 females) who completed the questionnaire distributed to 200 individuals with SCI (Response rate 33%). Inclusion criteria were as follows: (1) patients with SCI who were ≥18 years at the time consent was obtained and (2) those who consented to participate in this study. Bedridden patients with SCI were excluded from the study. This study was approved by the Ethical Committee for Epidemiology of Hiroshima University (approval number: E2022-0056) and conducted in accordance with the tenets of the Declaration of Helsinki. Informed consent was obtained from all the participants prior to the commencement of the study.

### Demographic characteristics

This survey was conducted in conjunction with two Japanese sports centers, one welfare center, and two hospitals. Posters were posted at each facility and requests were made to acquaintances to recruit the participants. A Google form, which has been used in several studies, was used to administer the questionnaire. The survey was fielded between November 1, 2022 and December 1, 2022. Information regarding age, height, weight, body mass index (BMI), age at which SCI occurred, sex, whether the injury was complete or incomplete, pressure ulcer status, marital status, living situation, occupation, non-sports exercise, time allotted for sports, time allotted for exercise, screen time, and type of para-sport in which they participate was collected. The respondents were asked to respond by defining sports as participation in some kind of community and exercise as physical activity by oneself to improve health.

### Outcome measures

#### Activities of daily living of individuals with SCI

The level of independence of individuals with SCI in their activities of daily living (ADL) was assessed using the Japanese version of the spinal cord independence measure-self report (JSCIM-SR). This tool is a self-reported version of the spinal cord independence measure, one of the most commonly used tools to assess ADL in individuals with SCI. The JSCIM-SR contains 17 items and is divided into three areas: self-care, breathing, and elimination management^15^^,^^16)^. The total score is 100 points, with higher scores assigned to items considered more important to achieve (such as urinary drainage management). Each item is scored on a scale of 2–9, considering the level of assistance needed to perform the activity and the presence or absence of assistive devices or environmental adjustments^17)^.

#### Physical activity

This study investigated physical activity using the International Physical Activity Questionnaire-Short Form (IPAQ-SF)^18)^. The IPAQ-SF comprises three questions regarding the days and duration of vigorous (8.0 metabolic equivalents [METs]), moderate (4.0 METs), and walking for more than 10 min (3.3 METs), as well as 1 question about daily sedentary time over the past 7 days. To accommodate the function of individuals with SCI who use wheelchairs, some questionnaire items were modified based on previous studies by Hurtig-Wennlöf et al.^19)^. These modifications included additional examples of vigorous and moderate activities, including wheelchair activities. The walking question was modified to include time spent walking, self-propelled wheeling, or equivalent light activities. Sedentary time was changed to inactive time. The IPAQ-SF score was calculated as total weekly physical activity (METs*min/week) using the IPAQ scoring instructions.

#### Mental health

The World Health Organization Five Well-Being Index (WHO-5) was used to measure subjective well-being. The WHO-5 has been used widely in research studies to validly measure well-being^20^^,^^21)^. Respondents were asked to rate how well the five statements applied to them over the past 14 days. The responses ranged between 0 (none of the time) and 5 (all the time). Raw scores ranged between 0 (absence of well-being) and 25 (maximal well-being). The Kessler screening scale for psychological distress (K6) was used to assess psychological distress among the participants^22)^. The K6 comprises six questions that assess depression and anxiety over the past month, with each question rated on a scale from 0 (never) to 4 (always). The total score ranges between 0 (no psychological distress) and 24 (severe psychological distress). The Japanese version of the K6 is used in research studies in Japan, and its efficacy has been demonstrated in previous studies^23)^.

#### Lifestyle habits

The health practice index (HPI) was used to evaluate lifestyle habits^24)^. The HPI is a useful tool that can be used to easily assess health-related lifestyle habits. It consists of seven questions regarding sleeping habits, breakfast consumption, snacking behavior, weight management, exercise habits, alcohol consumption, and smoking. The total score is 7 points, with higher scores indicating healthier lifestyle habits.

#### Severity of insomnia

The severity of insomnia was evaluated using the insomnia severity index—Japanese version (ISI-J), a valid tool for subjectively assessing and screening insomnia severity. Previous studies have demonstrated its reliability and validity^25)^. The ISI-J consists of seven questions, each scored on a 5-point scale ranging between 0 and 4 points. The total score ranges from 0 to 28 points, with higher scores indicating a higher subjective severity of insomnia. Respondents with an ISI score of ≥10 were considered at high risk for insomnia^26)^.

#### Relationships in the social community

The HRSC scale was used to assess the relationship between health and social communities^27)^. The HRSC scale, developed by Saito et al., consists of 11 items including participation in volunteer groups, sports groups, hobbies, study or cultural groups, skills teaching, community trust, community contribution, community attachment, emotional support received, emotional support provided, and instrumental support received. Respondents were asked to indicate the frequency or level of their involvement in each item. The total possible score is 11 points, which indicates the highest level of relationship in the social community.

#### Data analysis

Data analyses were conducted using SPSS version 28.0 for Mac (IBM Japan, Tokyo, Japan). The normality of data distribution was tested using the Shapiro–Wilk test. If the normality assumption was satisfied, the characteristics and outcome measures (including JSCIM-SR, IPAQ-SF, WHO-5, K6, HPI, HRSC, and ISI-J) were compared between individuals with SCI who participated in sports and those who did not use the unpaired t-test. The Wilcoxon rank-sum test was used if the normality assumption was not satisfied. The chi-square test was used for sex differences, pressure scores, marital status, living situation, occupation, and non-sport exercise. Pearson’s product–moment correlation coefficient was used to examine the relationship between HRSC and other outcome measures. Multiple regression analysis was then performed between HRSC and related variables. Results were reported as mean ± standard deviation (SD), and statistical significance was set at p <0.05. Sample size calculation was conducted using G*Power version 3.1 (Kiel University, Kiel, Germany), which indicated that 65 participants were required to achieve 80% power to test the primary result (α = 0.05, effect size = 0.15, number of predictors = 2).

## Results

**Fig. 1.**
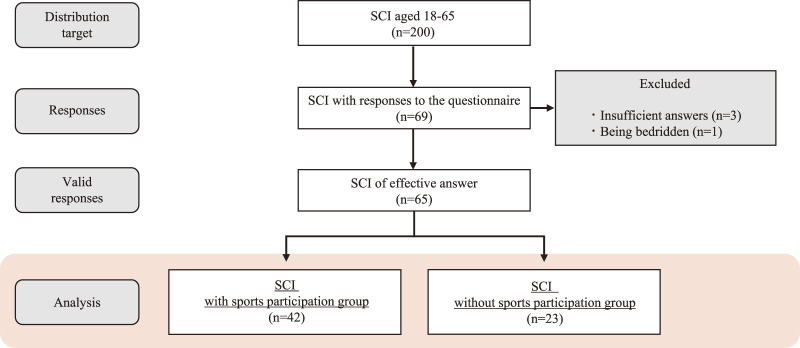
Study flow of this survey

Figure 1 shows the study flow for this survey. Responses were obtained from 69 of the 200 respondents. Three insufficient responses and one bedridden person were excluded, leaving a total of 65 respondents for analysis. The participants were divided into two groups based on whether they currently participate in sports or not. Table 1 shows the characteristics of individuals with SCI who did and did not participate in sports. No significant differences were observed in terms of age, height, weight, BMI, age at injury, sex differences, pressure scores, marital status, living situation, occupation, and non-sport exercise.

**Table 1.** Characteristics of SCI with and without sports participation

	SCI with sports participation	SCI without sports participation	p value
	Mean ± SD	Mean ± SD	
Age	42.5 ± 10.2	41.6 ± 8.5	0.724
Body height (m)	172.6 ± 7.2	171.92 ± 6.8	0.697
Body weight (kg)	65.0 ± 11.8	63.57 ± 9.5	0.613
BMI (kg/m^2^)	21.8 ± 3.6	21.5 ± 3.0	0.751
Age at injury	24.5 ± 8.6	22.7 ± 6.5	0.371
Sex, n (%)			0.496
Male	40 (95.2)	21 (91.3)	
Female	2 (4.8)	2 (8.7)	
SCI degree, n (%)			0.583
Complete	29 (69.0)	18 (78.3)	
Incomplete	13 (31.0)	5 (21.7)	
Pressure sores, n (%)			0.478
Once	32 (65.3)	19 (59.4)	
Twice or more	17 (34.7)	13 (40.6)	
Marital status, n (%)			0.307
Single	21 (50.0)	9 (39.1)	
Married	21 (50.0)	14 (60.9)	
Living situation, n (%)			0.487
Cohabiting or in a partnership	30 (71.4)	19 (82.6)	
Separated or divorced	12 (28.6)	4 (17.4)	
Occupation, n (%)			0.767
Company owner	4 (9.5)	1 (4.3)	
Company employee	18 (42.9)	11 (47.8)	
Contract employee	4 (9.5)	2 (8.7)	
Part-time	2 (4.8)	2 (8.7)	
Self-employed	2 (4.8)	0 (0)	
Professional athlete	1 (2.4)	0 (0)	
Unemployed	7 (16.7)	5 (21.7)	
Student	2 (4.8)	0 (0)	
Civil servant	2 (4.8)	1 (4.3)	
Real estate renter	0 (0)	1 (4.3)	
Non-sport exercise, n (%)			0.061
Yes	34 (81.0)	14 (60.9)	
No	8 (19.0)	9 (39.1)	

SCI, spinal cord injuries; SD, standard deviation; BMI, body mass index

**Fig. 2.**
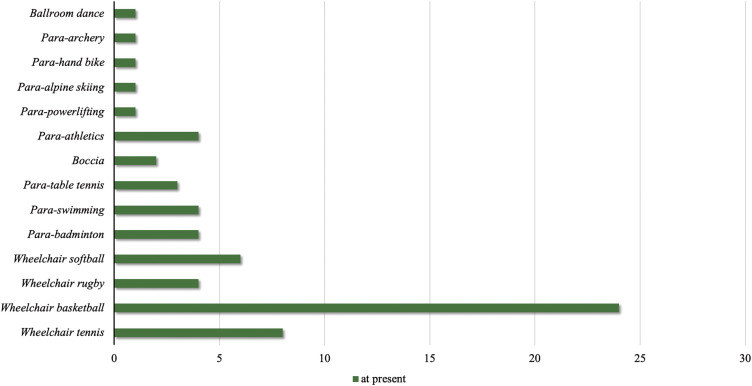
Types of para-sports in which spinal cord injury presently participate

Figure 2 shows the types of para-sports that individuals with SCI have participated. The most common sports individuals with SCI participated were wheelchair basketball (24), wheelchair tennis (8), and wheelchair softball (6), following that order. Individuals with SCI presently participating in sports had participated before in the following sports: wheelchair basketball (31), wheelchair tennis (18), and para-swimming (14). Among individuals with SCI that were not presently participating in sports, the most common sports in which they had participated were wheelchair basketball (10), wheelchair table tennis (7), and wheelchair rugby, boccia, and para-athletics (3 each), in that order.

A comparison of SCI indicator outcomes with and without sports participation is shown in Table 2. The time spent on sports, time spent doing exercise, IPAQ-SF, K6, and HRSC were significantly higher in individuals with SCI who participated in sports (p <0.01 or p <0.05). The WHO-5 was significantly lower in individuals with SCI who participated in sports (p <0.05).

**Table 2.** Comparison of indicator outcomes for SCI with and without sports participation

	SCI with sports participation	SCI without sports participation	p value
	Mean ± SD	Mean ± SD	
Time for sports (hour/year)	168.3 ± 100.0	0.0 ± 0.0	<0.001**
Time for exercise (hour/year)	142.7 ± 104.8	66.3 ± 74.4	0.003**
Time for smartphone (min/day)	830.5 ± 373.3	820.4 ± 426.2	0.922
JSCIM-SR	57.2 ± 14.7	48.6 ± 16.8	0.051
IPAQ-SF	4696.6 ± 6001.8	1184.8 ± 2012.4	0.001**
WHO-5	14.2 ± 5.6	17.1 ± 4.7	0.040*
K6	6.5 ± 4.9	3.3 ± 2.9	0.002**
HPI	4.2 ± 1.1	4.3 ± 1.3	0.878
ISI	7.5 ± 6.3	7.7 ± 6.0	0.903
HRSC	6.5 ± 2.2	5.3 ± 2.3	0.033*

*p <0.05; **p <0.01. p <0.05 and p <0.01 are shown in bold.

SCI, spinal cord injuries; SD, standard deviation; JSCIM-SR, Japanese version of the spinal cord independence measure-self report; IPAQ-SF, International Physical Activity Questionnaire-Short Form; WHO-5, The World Health Organization Five Well-Being Index; K6, Kessler screening scale for psychological distress; HPI, health practice index; ISI, insomnia severity index; HRSC, health-related social capital

Table 3 shows the relationship between HRSC and indicator outcomes in individuals with SCI who did and did not participate in sports. SCI with sports participation showed a significant positive correlation between HRSC and HPI (r = 0.336, p <0.030). By contrast, SCI with sports participation showed a significant negative correlation between HRSC and ISI (r = −0.308, p <0.048).

**Table 3.** Relationship between indicator outcomes and HRSC in SCI with and without sports participation

Variables	HRSC in SCI with sports participation		HRSC in SCI without sports participation	
	r	p	r	p
JSCIM-SR	0.057	0.726	0.081	0.741
IPAQ-SF	0.066	0.677	0.152	0.490
WHO-5	0.201	0.201	0.065	0.769
K6	−0.089	0.575	−0.026	0.905
HPI	0.336	0.030*	−0.278	0.200
ISI	−0.308	0.048*	−0.154	0.483

*p <0.05. p <0.05 is shown in bold. Each variable was tested by the Pearson correlation coefficient.

HRSC, health-related social capital; SCI, spinal cord injuries; JSCIM-SR, Japanese version of the spinal cord independence measure-self report; IPAQ-SF, International Physical Activity Questionnaire-Short Form; WHO-5, The World Health Organization Five Well-Being Index; K6, Kessler screening scale for psychological distress; HPI, health practice index; ISI, insomnia severity index

Multiple regression analyses of HPI and ISI for HRSC in individuals with SCI who did and did not participate in sports are shown in Table 4. SCI with sports participation and high HRSC showed an association with HPI. In post hoc power analysis, the multivariate regression model for the association between HPI and ISI for SCI with sports participation of high HRSC demonstrated sufficient power (1 err prob = 0.976).

**Table 4.** Multiple regression analysis of HPI and ISI for HRSC in SCI with and without sports participation

Variables	HRSC in SCI with sports participation					HRSC in SCI without sports participation				
	R2	β	p	95% CI interval		R2	β	p	95% CI interval	
				Lower	Upper				Lower	Upper
HPI	0.183	−0.300	0.047*	0.008	1.216	0.145	−0.369	0.108	−1.438	0.153
ISI		−0.267	0.075*	−0.195	0.010		−0.276	0.222	−0.285	0.070

*p <0.05. p <0.05 is shown in bold. Explanatory variables were HPI and ISI. β: standardized partial regression coefficient.

HPI, health practice index; ISI, insomnia severity index; HRSC, health-related social capital; SCI, spinal cord injuries; CI, confidence interval

## Discussion

This was the first study to clarify the characteristics and outcomes of individuals with SCI who did and did not participate in sports, with a particular focus on the role of HRSC. The results yielded several significant findings, obtained interesting findings on the potential effects of sports involvement on various health-related indicators, and the relationship of HRSC and HPI in SCI with sports participation.

Individuals with SCI engaged in sports demonstrated significantly higher scores for time spent on sports and exercise and the IPAQ-SF, indicating greater overall physical activity levels. These findings align with those of previous research highlighting the positive impact of sports involvement on physical activity among individuals with disabilities, including those with SCI^28^^,^^29)^. Sports participation provides the opportunity to engage in meaningful physical activities and contributes to the overall well-being of an individual^30)^. However, it is noteworthy that individuals with SCI who participated in sports showed significantly higher psychological distress, as indicated by higher K6 scores. Moreover, this was confirmed by significantly lower WHO-5 scores. Though the results showed a different trend from previous studies, it has been previously discussed that mental health is related to quality of life^11^^,^^31)^. The environment surrounding SCI has a significant impact on the quality of life, although the supporting framework is also a major factor in the satisfaction of SCI with their lives^31^^,^^32)^. There were no significant differences between the demographics of individuals with SCI who participated in this study in whether they participated in sports or not. One possible explanation suggests that psychological aspects associated with sports involvement, such as competition, performance pressure, and adjustment challenges, may contribute to increased distress among individuals with SCI^33^^,^^34)^.

Interestingly, individuals with SCI who participated in sports had significantly higher HRSC scores. This finding suggests that engagement in sports activities may foster the development of HRSC in individuals with SCI. HRSC encompasses the resources, support, and networks available to individuals within their social environment, particularly regarding health and well-being^9)^. The results of significantly higher HRSC scores for individuals with SCI who participate in sports suggest that sports may contribute to the formation of social connections and the development of social support, which may improve the health outcomes of individuals with SCI. Furthermore, the positive correlation between HRSC and HPI among individuals with SCI who participate in sports suggests that HRSC may play a significant role in shaping the lifestyle factors that are captured by the index^35^^,^^36)^. In addition, access to social support networks within the social environment for individuals with SCI may facilitate a better lifestyle, as significant negative correlations were identified in the ISI. The resulting sleep quality may reduce insomnia and its associated side effects^36^^,^^37)^. This indicates that the availability of social capital may influence lifestyle choices, behaviors, and overall well-being of individuals with SCI who actively participate in sports.

Our findings suggest that sports participation among individuals with SCI positively affects physical activity levels and may contribute to the development of HRSC. However, further investigations are required to better understand the psychological well-being implications of sports participation in this population, emphasizing the importance of providing appropriate psychological support alongside sports participation.

This study had a few limitations. First, this was a cross-sectional study, which may have limited our ability to establish a causal relationship among HRSC, other factors, or outcomes of participation in sports in individuals with SCI. Second, with a limited subject scope and not enough participants, and since the study population comprised mostly of males, it did not provide an overall view of the SCI population. However, the strength of this study was that it examined the characteristics and outcomes of individuals with SCI who participated in sports, the potential impact of sports participation, and the benefits of HRSC.

## Conclusion

This study determined whether HRSC and other outcomes differed depending on whether individuals with SCI did or did not participate in sports activities. Furthermore, our study showed that individuals with SCI who participated in sports had higher HRSC, and their HRSC was associated with HPI and ISI. Sports participation in individuals with SCI may contribute to the development of HRSC and influence lifestyle choices. These results provide important data that may help encourage sports participation in individuals with SCI.

## Acknowledgments

The authors thank all the study participants.

## Funding

This work was supported by Japanese Society of Physical Therapy (JSPT2022-gen083).

## Conflicts of Interest

The authors declare that they have no conflicts of interest.
